# Mapping the lymphatic system across body scales and expertise domains: A report from the 2021 National Heart, Lung, and Blood Institute workshop at the Boston Lymphatic Symposium

**DOI:** 10.3389/fphys.2023.1099403

**Published:** 2023-02-06

**Authors:** Dhruv Singhal, Katy Börner, Elliot L. Chaikof, Michael Detmar, Maija Hollmén, Jeffrey J. Iliff, Maxim Itkin, Taija Makinen, Guillermo Oliver, Timothy P. Padera, Ellen M. Quardokus, Andrea J. Radtke, Hiroo Suami, Griffin M. Weber, Ilsa I. Rovira, Selen C. Muratoglu, Zorina S. Galis

**Affiliations:** ^1^ Department of Surgery, Division of Plastic and Reconstructive Surgery, Beth Israel Deaconess Medical Center, Harvard Medical School, Boston, MA, United States; ^2^ Department of Intelligent Systems Engineering, Luddy School of Informatics, Computing, and Engineering, Indiana University Bloomington, Bloomington, IN, United States; ^3^ Institute of Pharmaceutical Sciences, Swiss Federal Institute of Technology (ETH) Zürich, Zürich, Switzerland; ^4^ MediCity Research Laboratory, University of Turku, Turku, Finland; ^5^ VISN 20 Mental Illness Research, Education and Clinical Center (MIRECC), VA Puget Sound Healthcare System, Department of Psychiatry and Behavioral Science, Department of Neurology, University of Washington School of Medicine, Seattle, WA, United States; ^6^ Center for Lymphatic Imaging and Interventions, Perelman School of Medicine, University of Pennsylvania, Philadelphia, PA, United States; ^7^ Department of Immunology, Genetics and Pathology, Uppsala University, Uppsala, Sweden; ^8^ Center for Vascular and Developmental Biology, Feinberg School of Medicine, Feinberg Cardiovascular and Renal Research Institute, Northwestern University, Chicago, IL, United States; ^9^ Department of Radiation Oncology, Massachusetts General Hospital and Harvard Medical School, Boston, MA, United States; ^10^ Lymphocyte Biology Section and Center for Advanced Tissue Imaging, Laboratory of Immune System Biology, National Institute of Allergy and Infectious Diseases (NIAID), National Institutes of Health (NIH), Bethesda, MD, United States; ^11^ Department of Clinical Medicine, Australian Lymphoedema Education, Research and Treatment Centre, Macquarie University, Sydney, NSW, Australia; ^12^ Division of Cardiovascular Sciences, National Heart, Lung, and Blood Institute (NHLBI), National Institutes of Health (NIH), Bethesda, MD, United States

**Keywords:** lymphatic disease, lymphedema, lymphatic research, mapping, lymphatic anatomy

## Abstract

Enhancing our understanding of lymphatic anatomy from the microscopic to the anatomical scale is essential to discern how the structure and function of the lymphatic system interacts with different tissues and organs within the body and contributes to health and disease. The knowledge of molecular aspects of the lymphatic network is fundamental to understand the mechanisms of disease progression and prevention. Recent advances in mapping components of the lymphatic system using state of the art single cell technologies, the identification of novel biomarkers, new clinical imaging efforts, and computational tools which attempt to identify connections between these diverse technologies hold the potential to catalyze new strategies to address lymphatic diseases such as lymphedema and lipedema. This manuscript summarizes current knowledge of the lymphatic system and identifies prevailing challenges and opportunities to advance the field of lymphatic research as discussed by the experts in the workshop.

## Introduction

The lymphatic vasculature is comprised of a vast network of vessels in all tissues of the body that converge to transport lymph away from tissues to the blood in order to maintain extracellular fluid homeostasis and provide critical immunologic trafficking. This traditional paradigm of passive transport of lymph fluid has been updated by cellular and molecular characterization of lymphatic vascular development. Lymph production and flow is critical for removal of interstitial fluid from tissues to prevent tissue edema. Our greatly improved understanding of the function of the lymphatic vasculature has revealed new roles in health and disease.

The scope of lymphatic disease research is broad and includes, but is not limited to, investigating lymphedema, lipedema, chylous leak disorders, lymphatic malformations, and protein losing enteropathies (Dori et al., 2016; Itkin et al., 2017; O'Leary et al., 2021). While some of these may not be commonly occurring conditions, recent investigations of the lymphatic system anatomy, using newly described lymphatic imaging methods, have led to observations that lymphatic anatomy variations may be the culprit in highly prevalent and morbid disease states. For example, congestive heart failure affects more than six million Americans annually (Virani et al., 2020). Lymph production and flow is critical for removal of interstitial fluid from tissues to prevent tissue edema. Inadequate lymph flow caused by variants in lymphatic anatomy may contribute to abnormal patterns of lymph flow in certain vital organs including the heart, contributing to the overall pathophysiology of tissue- and organ-specific congestion in heart failure (Itkin et al., 2021). Similarly, secondary lymphedema, a disease with no known cure, is considered one of the most significant cancer survivorship morbidities in the United States. Breast cancer-related lymphedema results from obstruction or disruption of the lymphatic system associated with cancer treatment. Variations in peripheral lymphatic anatomy of the upper arm may explain why certain women undergoing breast cancer treatment develop breast cancer related lymphedema and others do not (Johnson et al., 2020a; Johnson et al., 2020b). Further, the success of rerouting and reconstruction of lymphatic flow may be dependent on the anatomic variations in lymphatic drainage that could predispose, or protect, an individual from the development of lymphedema. Despite these observations, the most respected compendium of lymphatic anatomy knowledge, the textbook “Anatomy of the Human Lymphatic System” by Professor H. Rouvière, is significantly outdated, having been published in 1938 (Rouvière, 1938). There is an unmet need to update our knowledge of lymphatic anatomy utilizing modern methods of functional lymphatic imaging.

The 2021 National Heart, Lung, and Blood Institute Workshop *Yet to be Charted: Mapping the Lymphatic System Across Body Scales and Expertise Domains* at the Boston Lymphatic Symposium identified major knowledge gaps in lymphatic anatomy and lymphatic biomolecular signatures as major barriers needing to be addressed to accelerate advancement of medical management of lymphatic diseases. Research opportunities and ongoing efforts to address these gaps were discussed and are presented in this report.

### Lymphatic anatomy and the knowledge gaps explained

The lymphatic vasculature consists of a network of thin-walled, blind-ended, highly permeable initial lymphatics or lymphatic capillaries which first drain into pre-collecting lymphatic vessels, merging into larger secondary collecting lymphatics. The valves of the collecting lymphatics control the unidirectional transport of lymph back to the blood circulation. Lymph is transferred to pre-nodal collecting lymphatics, also called afferent lymphatics, leading to lymph nodes. The lymph exits lymph nodes through post-nodal collecting lymphatics, eventually draining into the thoracic duct and the right lymphatic duct, which, in turn, discharge lymph into the large veins at the base of the neck. More in-depth reviews provide a detailed understanding of the structure, anatomy, development, and embryogenic origins of the lymphatic system (Breslin et al., 2018; Oliver et al., 2020). Despite greatly improved understanding of the lymphatic vasculature, current knowledge of the map of the lymphatic system is far from complete.

The lack of clinically relevant knowledge of the human lymphatic system, in comparison to the remainder of the vascular system, is due to several reasons. First, while lymphatic capillaries are significantly larger than blood capillaries, the remainder of the lymphatic vasculature is significantly smaller than the major arteries and thus are challenging to appreciate with the naked eye during surgery or even with existing clinical imaging methods (Singhal et al., 2019). Second, damage to the arterial and/or venous vasculature results in obvious bleeding while lymphatic damage results in leakage of a clear fluid which easily goes unnoticed by anatomists, surgeons, or radiologists. Third, lymphatic anatomy, similar to venous anatomy, is extraordinarily variable. Embryologically, the lymphatic system derives from multiple developmental origins to form primitive lymphatic structures, which then fuse together to build a lymphatic network (Ulvmar and Mäkinen, 2016; Oliver et al., 2020). The fusion process can be altered and affected at different stages of embryological development leading to significant variability. Fourth, the size and variability of the lymphatic system explained above have led to challenges in clinical diagnostic imaging since consistent and reproducible introduction of contrast into lymphatic vessels is difficult. To further complicate matters, while the blood vascular system can be investigated with dye or radiocontrast perfusion in cadavers, valvular structures with millimeter-long intervals in the lymphatic vessel prevent retrograde injection from a proximal site. Therefore, a contrast agent must be introduced distally where the lymphatic system is the smallest and most variable. With limited availability of clinical imaging of the lymphatic system, our knowledge of the anatomy is further hindered. Finally, our current knowledge about gross lymphatic anatomy is attributed to findings by anatomists from a prior century (Sappey, 1874). They used mercury injection to demonstrate the lymphatic system, but this method fell out of favour due to mercury toxicity. Thus, gross lymphatic anatomy has not been updated for more than a century. As our knowledge of macro-lymphatic anatomy remains rudimentary, our omic data of human lymphatics similarly lags behind.

### Knowledge gaps in lymphatic biomolecular signatures explained

While actual translation of omic data into significant clinical interventions is in the nascent phase, the promise of multi-omic data in driving diagnosis, prognosis, and providing targets for intervention is an area of intense study. Therefore, it is only to be expected that a tremendous effort has been placed on obtaining the biomolecular signatures of the human cardiovascular system (Leon-Mimila et al., 2019). Surprisingly, the characterization of human lymphatic vessels using novel techniques, such as spatial tissue profiling and single cell sequencing has started only recently. Much of our in-depth knowledge of cellular and biomolecular lymphatic vessel signatures to date comes from animal-derived studies (Kalucka et al., 2020). However, despite their genetic similarity to humans, mouse models are criticized for their failure to accurately mimic human disease phenotypes and their inaccurate portrayal of the human condition for a multitude of reasons including: i) less individual genetic variations, ii) inability to faithfully recapitulatecomplex nutrition- and lifestyle-associated changes in lymphatic structure and function, and iii) basic biomechanical properties such as being bipedal, i.e., the upright postural position of the human body can profoundly impact anatomy and function of the lymphatic system (Gashev and Zawieja, 2010).

In a self-perpetuating manner, the challenges of visualizing the lymphatic system either grossly or with imaging and gaps in biomolecular signatures has been further exacerbated by insufficient coverage of this topic in medical education and training (Granger et al., 2004). Such knowledge gaps amongst clinicians have significant implications on patient care as, for example, early accurate detection of lymphatic disease can improve clinical outcomes (Blome et al., 2013; Rockson, 2018). Furthermore, left untreated, lymphatic dysfunction can increase a patient’s risk for cardiovascular and chronic inflammatory diseases and significantly impact their emotional and mental wellbeing (Fu et al., 2013; Mortimer and Rockson, 2014).

## Lymphatic anatomy

### Clinical anatomy and imaging today

**FIGURE 1:**
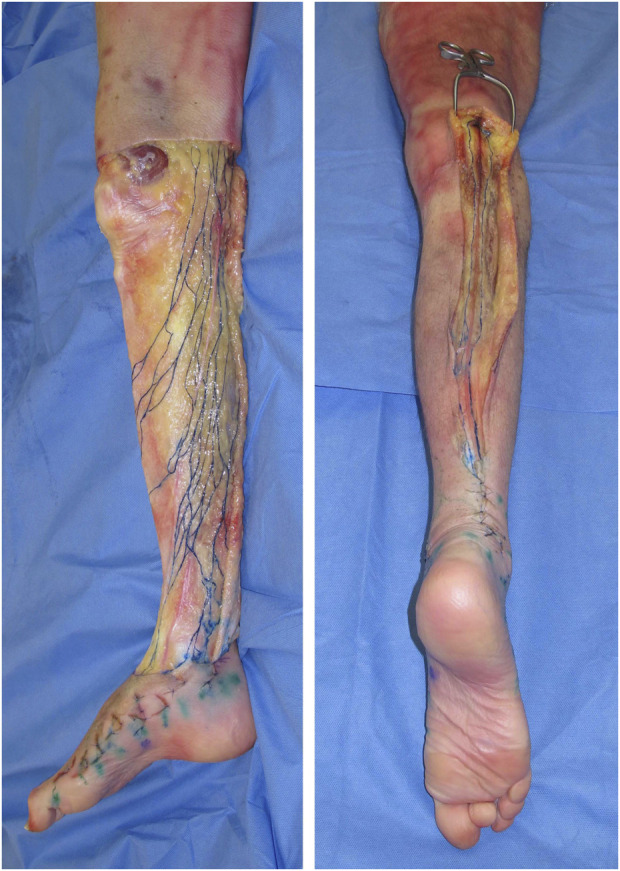
The lower extremity of a cadaver injected with dye into the superficial lymphatic vessels shows the medial pathway (left) and posterior pathway running along the small saphenous vein (right).

**FIGURE 2:**
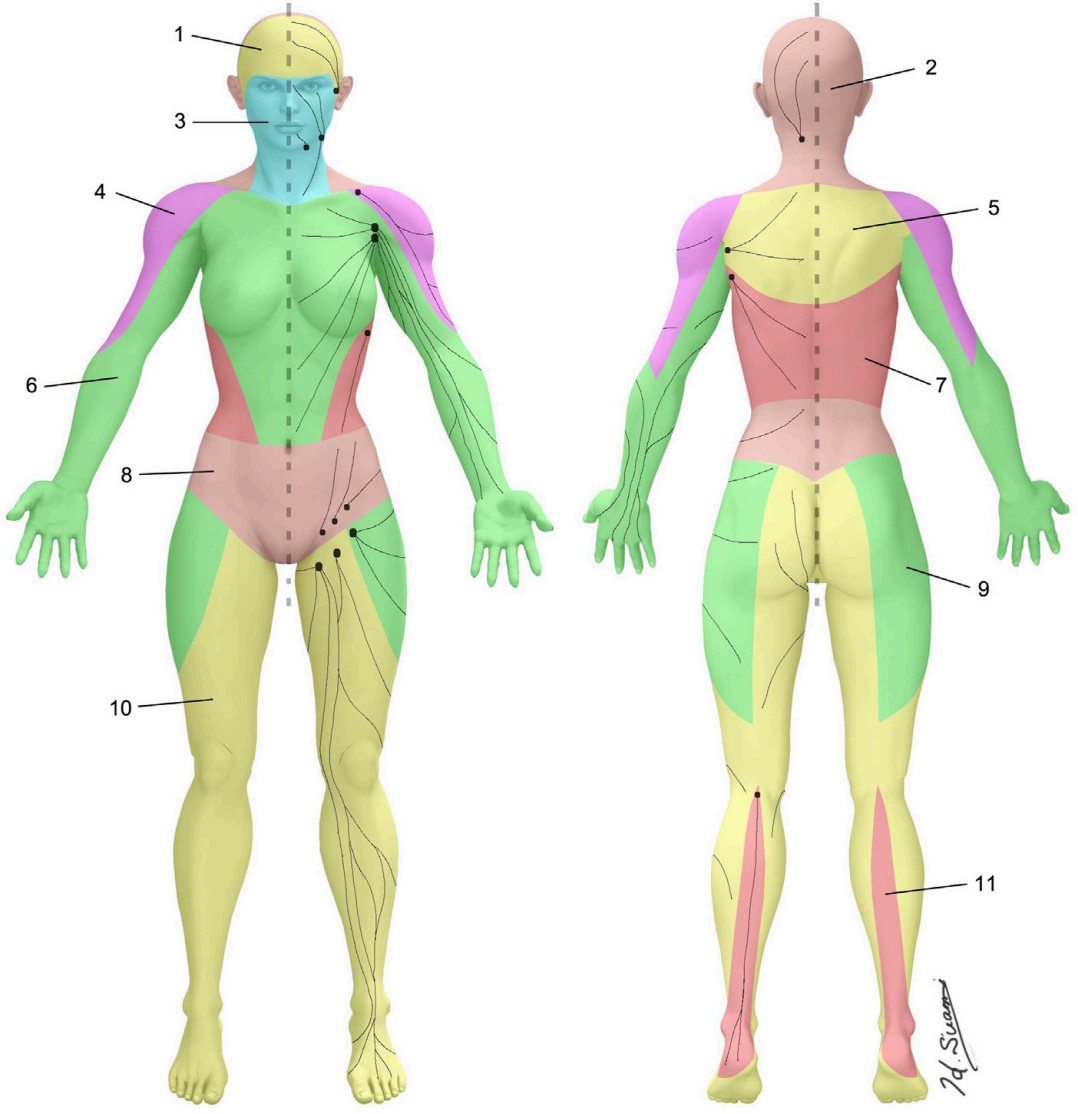
Lymphosomes of the body. The lymphatic territories are demarcated according to their corresponding lymphatic basins: 1. Temporal, 2. Occipital, 3. Superficial cervical, 4. Subclavicular, 5. Subscapular, 6. Lateral axillary, 7. Pectoral, 8. Superior inguinal, 9. Lateral inguinal, 10. Inferior inguinal, 11. Popliteal.

Traditionally, our understanding of clinical anatomy begins in the gross anatomy laboratory. As the challenges of gross anatomic dissection of the lymphatic system have been previously described, new techniques in anatomic dissection have been recently introduced. Specifically, a microinjection technique utilizing hydrogen peroxide now allows for reliable simultaneous identification and dilation of the lymphatic channels which is accomplished when oxygen bubbles are absorbed into the lymphatic vessels (Suami et al., 2007). Overcoming the challenges of size, these vessels are now more easily directly cannulated, allowing for reliable and reproducible injections of a contrast agent (Suami and Scaglioni, 2018) (Figure 1). As our ability to visualize lymphatics in cadavers has improved, so has our understanding of lymphatic anatomy. The lymphatic system is the third vascular system and therefore it would not be surprising to find parallels between the blood and lymphatic vasculature. In 1987, the concept of angiosomes was introduced, where all the soft tissue from the bone to the skin was divided into three-dimensional vascular territories and each territory could be traced to a source artery and vein (Taylor and Palmer, 1987). Today, with the improvement of lymphatic visualization in cadavers utilizing hydrogen peroxide, it has been noted that lymph nodes reliably drain a defined skin territory or “lymphosome” (Suami et al., 2005). The lymphosome chart can provide a beginning framework from which normal lymphatic anatomy can be further detailed (Figure 2). Similar concepts were also introduced in the 1700s by Mascagni (Mascagni, 1787).

**FIGURE 3:**
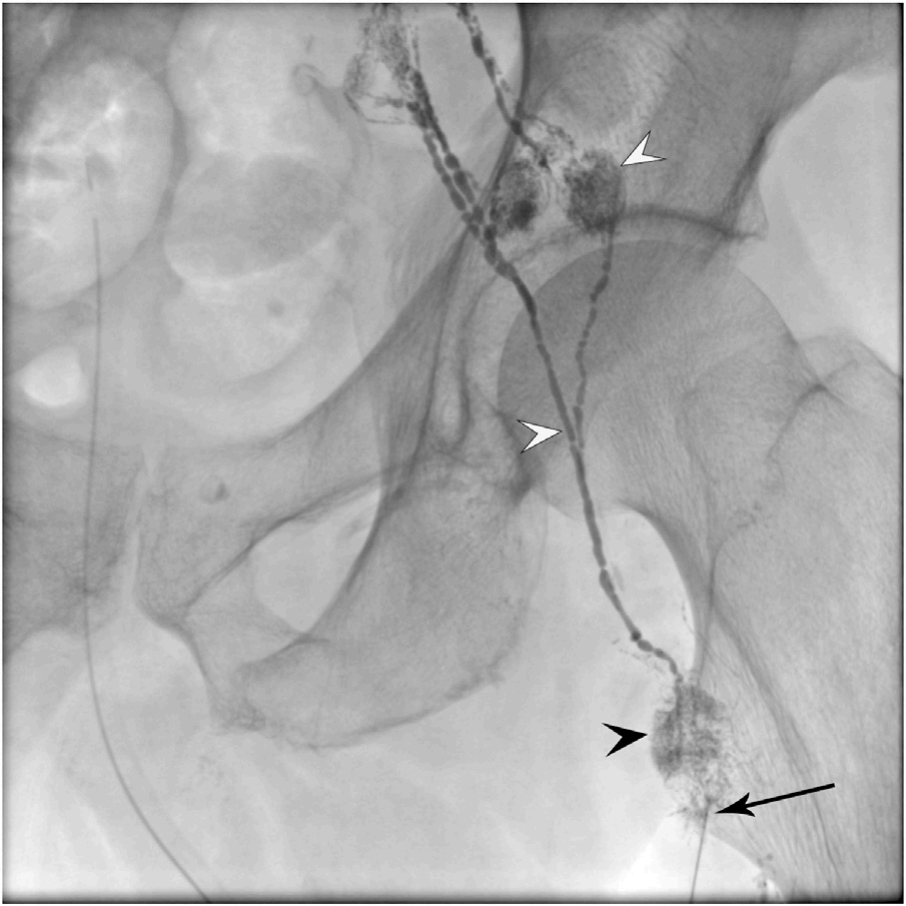
The fluoroscopic image of the intranodal lymphangiography that is performed by placing a needle (arrow) inside the inguinal lymph node (black arrowhead) and injecting oil based iodinated contrast. The efferent lymphatics and distal lymph nodes (white arrowheads) are opacified.

Advances in understanding of structural and physiological characteristics of the lymphatic vasculature have guided improvements in the clinical imaging of the lymphatic system. The two main clinical lymphatic imaging techniques for the last 70 years were pedal lymphangiography and lymphoscintigraphy. However, these techniques have significant drawbacks. In order to overcome the drawbacks, a newer approach has been developed by introducing contrast (oil and water based iodinated as well as gadolinium) through a needle into the lymphatic system by accessing organs rich with lymphatic vessels such as lymph nodes or the liver. This technique is often referred to as an interstitial injection. This approach has led to the development of innovative imaging techniques utilizing fluoroscopy such as intranodal lymphangiography, liver lymphangiography, mesenteric lymphangiography as well as dynamic contrast enhanced magnetic resonance lymphangiography and computed tomography lymphangiography (Nadolski and Itkin, 2012; Chavhan et al., 2017; Lee et al., 2018; Itkin et al., 2020; Lee et al., 2022). These modalities allow for excellent visualization of most parts of the lymphatic system (Figure 3). In turn, these new modalities have provided better insights into the lymphatic system structure and lymph flow dynamics essential for lymphatic disease mechanisms. For instance, these new imaging modalities have already demonstrated that anatomical variants of the lymphatic system often play a crucial role in the pathophysiology of a variety of diseases. These findings underscore the importance of creating a body of knowledge that details normal lymphatic anatomy and, subsequently, the range of the lymphatic anatomical variants (Singhal et al., 2019).

### Lymphatic anatomy and disease

Lymphedema is the most common of all the lymphatic disorders and is considered one of the most significant cancer survivorship issues in the United States. Despite its relative prevalence, it remains unclear why certain cancer patients develop this condition following treatment while others are spared. Focusing specifically on breast cancer related lymphedema (BCRL), the top 3 risk factors include an axillary lymph node dissection, regional lymph node radiation, and a Body Mass Index (BMI) > 30. However, despite these known risk factors, only 1 in 3 women with all three risk factors will develop lymphedema. Utilizing the modern imaging technique of indocyanine green (ICG) lymphography to obtain real-time scans of the upper extremity peripheral lymphatic systems prior to axillary lymph node dissection, significant variations in the superficial lymphatic anatomy have been recently demonstrated. Anatomists had previously postulated that collateral lymphatic pathways of the upper extremity, or a pathway that avoids the axilla, may be protective against BCRL as the lymphatic flow would divert away from the axilla following dissection. Modern ICG imaging has recently confirmed the presence of two variants of a specific collateral pathway, the Mascagni–Sappey (M-S) pathway, which is hypothesized to decrease an individual’s risk of BCRL development (Singhal et al., 2019). Identification of these anatomic variants prior to cancer treatment will allow tumor boards, and patients themselves, to consider this information during treatment decision-making and also allow for the implementation of both surgical and non-surgical strategies for lymphedema prevention in cancer patients. Of note, separate preventative efforts are currently underway to preserve critical lymphatics at the time of nodal extirpation. For example, the concept of axillary reverse mapping, i.e., preservation of arm lymphatics during axillary lymph node dissection, is actively being studied (NCT03927027) (Klimberg, 2008; Tummel et al., 2017).

Centrally, lymphatic variants have been discovered to be the root cause of certain pulmonary disorders. Specifically, pulmonary lymphatic disorders (PLD) are a diverse group of conditions characterized by the presence of abnormal lymphatic tissue in the chest and are often accompanied by chylous leaks, such as non-traumatic chylothorax, plastic bronchitis and chyloptysis. PLD is associated with a variety of conditions including congenital heart disease, Noonan syndrome, and lymphatic malformations. The understanding of PLD has been limited due to lack of robust central lymphatic imaging. Recent development of the intranodal lymphangiography and Dynamic Contrast Enhanced MR lymphangiography demonstrated the presence of abnormal lymphatic pathways from the thoracic duct and/or retroperitoneum into lung parenchyma, termed abnormal pulmonary lymphatic flow (PLF) (Gurevich et al., 2021). The abnormal PLF represents a congenital anatomical lymphatic variant that can be asymptomatic or present clinically as early as a neonatal chylothorax or adult plastic bronchitis. The triggers for the clinical presentation vary and can be explained by an increase in lymphatic flow in patients with congenital heart disease resulting in congestion of the lymphatic system or obesity in patients with non-cardiac plastic bronchitis. Recent developments in minimally invasive techniques involving catheterization of the thoracic duct as well as interstitial lymphatic embolization allows for correction of the abnormal lymphatic flow, a promising treatment option for these conditions (Itkin and Nadolski, 2018).

### Research gaps and opportunities

It is incumbent on the lymphatic community to update our current compendium of normal lymphatic anatomy utilizing the most modern dissection, *in vivo* imaging, and knowledge management modalities.

The lymphatic system has an intricate superficial and deep vasculature. Our inability to reliably image the deep lymphatic system, and importantly, potential connections between the deep and superficial systems, remains a significant limitation in creating a map of the peripheral lymphatic system. Even the most modern imaging methods such as ICG lymphography that allows precise mapping of the superficial peripheral lymphatic system still have significant limitations such as its shallow depth of penetration of less than 2 cm (Unno et al., 2007). The greater depth of penetration of nuclear lymphoscintigraphy, considered the current gold standard technique for lymphatic imaging (Sage and Gozun, 1958), comes at the expense of resolution, the two-dimensional low-resolution images do not allow elucidation of high-resolution lymphatic anatomy.

Promising advances in magnetic resonance lymphography and photoacoustic images may soon help bridge this crucial gap. For advancing the understanding of the lymphatic vasculature, further research not only on the anatomy, but also on lymphatic flow will be critical to understand how abnormal central lymphatic system flow, such as PLF may play a role in serious conditions, including congestive heart failure and pulmonary alveolar proteinosis.

Additionally, all the information will need to be systematically incorporated and organized into a comprehensive, publicly available, updatable, online reference library of the normal lymphatic vasculature anatomy. As an initial step towards creating such a map, more than 900 entries related to lymphatic vessels and nodal stations have been extracted from the 1938 textbook by Rouvière and conceptually organized using a present-day approach, the “Miro Map”. The lymphatic vasculature map is available at a publicly accessible website^1^. Further efforts will be needed to make such information compatible with other ongoing mapping efforts focused on human blood vasculature, the lymphatic system, and other organs (Börner et al., 2021).

One of the most important parts of the efforts to map the normal human lymphatic vasculature across the scales of the human body will be to discover, catalogue, and explain the commonly shared clinical level features and the variants of the lymphatic anatomy in normal individuals. The mapping of anatomical variants remains a priority in order to better understand the foundation of several lymphatic disease pathophysiology. Taking a page from the current efforts to map the human blood vasculature^2^, that leveraged the Uber-anatomy ontology (UBERON) (Mungall et al., 2012) and the Foundational Model of Anatomy (FMA) (Noy et al., 2004), indicates many challenges are lying ahead, including individual normal variations, inconsistencies across organs, and changes across the lifespan, that will require collaborations across expertise domains. The review process has included identifying adult human blood vessels, correcting and standardizing vessel names, adding missing vessels, recording the organs or body regions associated with each vessel, and linking vessels to endothelial cell types and biomarkers. The development of the blood vasculature map has required close collaborations between experts with complementary expertise, including computer science and domain experts in vascular pathology, vascular biology, and vascular surgery.

Higher resolution investigations of cellular composition, organization, and biomolecular markers of the lymphatic system are also needed to understand the basis for normal lymphatic anatomy and variants, as well for the clinical management of lymphatic conditions.

## Advances in lymphatic cellular and biomolecular signatures in health and disease

While the etiology of primary lymphedema can often be clearly ascertained to genetic mutations, such as in Milroy’s disease where hereditary inactivating mutations in vascular endothelial growth factor (VEGF) receptor-3 (VEGFR-3) have been implicated, the underlying factors of secondary lymphedema development are less well understood. A variety of experimental models have been employed to study lymphedema development, including surgically disconnecting the superficial and deep lymphatic vessels leading to oedema formation in the tail extremity, or in the mouse paw after popliteal lymph node dissection. While both represent acute lymphedema models that spontaneously resolve over time with little fibrosis or adipose tissue deposition, they help point to cellular and molecular mechanisms that may be at play. For instance, researchers were able to identify that prolonged T helper two biased immune responses regulates the pathology of the response by promoting tissue fibrosis, inhibiting formation of collateral lymphatics, decreasing lymphatic vessel pumping capacity, and increasing lymphatic leakiness (Avraham et al., 2013).

Lipedema is a chronic adipose tissue disorder characterized by the disproportional subcutaneous deposition of fat and is commonly misdiagnosed as lymphedema or obesity. Compared to age and BMI matched patients, lipedema patients have increased systemic levels of VEGF-C, cholesterol, and triglycerides (Suami et al., 2005; Suami and Scaglioni, 2018). However, they do not seem to have morphological changes of lymphatic vessels nor differences in lipid composition of adipose tissue (Felmerer et al., 2020). Recent findings indicate, though, that lipedema patients have a distinct cytokine profile and increased metabolic activity of the stromal-vascular fraction containing adipose-derived stem cells, macrophages, and endothelial cells, among other differences (Wolf et al., 2021; Suami, 2022).

### Revealing connections between specific biomolecular signatures and functions of the lymphatic vasculature across the body

We have gained an increased appreciation of the important contributions of the lymphatic vasculature to the normal function of various organs, precipitating or helping with recovery from other medical conditions. Lymphatic vessels show remarkable plasticity and heterogeneity, reflecting their functional specialization to control the tissue microenvironment (Oliver and Srinivasan, 2010; Petrova and Koh, 2018; Oliver et al., 2020; Petrova and Koh, 2020). For instance, in the last few years it has been shown that restoring cardiac lymphatics might serve as a therapeutic target to promote recovery of cardiac function after ischemic heart injuries. Cardiac lymphatics form a vascular network that helps regulate and maintain fluid balance and immune surveillance in the heart, two key features observed to be at play during and after myocardial infarction (MI) (Brakenhielm and Alitalo, 2019). Stimulation of lymphangiogenesis in the ischemic areas by administration of the lymphangiogenic growth factor VEGF-C following ischemic injury greatly improved cardiac function and adverse remodeling in several animal models (Klotz et al., 2015; Vuorio et al., 2017; Brakenhielm and Alitalo, 2019) suggesting it as a new approach for the treatment of heart diseases. In addition, similar to blood endothelial cells (BECs) that produce tissue-specific molecules that participate in organ repair and regeneration, LECs are also capable of secreting paracrine factors such as growth factors, cytokines, and chemokines. Until recently, the few identified lymphoangiocrine signals were known to participate in the regulation of immune responses, especially in lymph nodes. Recent results provided new interpretations about the role of lymphatics in cardiac repair, as another novel functional role of cardiac-associated lymphatics was identified. Specifically, LECs-derived lymphoangiocrine factors, including the key molecule reelin, have been identified as key players in cardiac growth and repair (Liu et al., 2020). Maintenance of proper cardiac lymphatic function and the role of lymphatics in pathological conditions of the heart is a widely debated topic. A recent in-depth review of this topic has recently been published.

Over the last 10 years, the identification and characterization of venous sinus-associated lymphatic vessels embedded within the brain’s dura mater that drain solutes from the cerebrospinal fluid (CSF) and central nervous system (CNS) has allowed a dramatic revision of our understanding of brain fluid dynamics and waste clearance (Aspelund et al., 2015; Louveau et al., 2015). An integrated description of the meningeal lymphatic system and glymphatic system (Iliff et al., 2012; Iliff et al., 2013a; Xie et al., 2013), a brain-wide network of perivascular pathways that supports fluid and solute exchange between the CSF and CNS interstitial compartments, has begun to emerge. These new results demonstrate the existence of a complex and integrated system of interstitial fluid (ISF) and CSF drainage pathways and their role in cerebral drainage. Glymphatic exchange and waste clearance driven by arterial pulsation (Iliff et al., 2013b; Mestre et al., 2018b) are dependent upon the astroglial water channel aquaporin-4 (AQP4) (Iliff et al., 2012; Mestre et al., 2018a), and is more rapid in the sleeping compared to the waking brain (Xie et al., 2013; Eide et al., 2021). The clearance of solutes from the CNS to the cervical lymphatic drainage are dependent upon perivascular glymphatic exchange (Goodman and Iliff, 2020), while perivascular solute exchange and meningeal lymphatic drainage appear to be counter-regulated by the circadian cycle with more rapid perivascular exchange occurring during sleep-associated periods and more rapid meningeal lymphatic drainage occurring during wake-associated periods (Hablitz et al., 2020). These findings suggest that the integrated and sequential function of perivascular glymphatic exchange and meningeal lymphatic drainage appears to support the processes of waste clearance and peripheral immune surveillance, classical lymphatic functionswithin the CNS (Louveau et al., 2017). It is noteworthy that the variability in lymphatic anatomy observed peripherally thus far has not been described in the cases of perivascular glymphatic networks and meningeal lymphatic vasculature. It is likely that this is the result of the recency of the description of these structures (beginning in 2012–2015) and the paucity of studies of their anatomy and function in human clinical populations, rather than any peculiar uniformity unique to these cranial structures. Future work in this emerging biology is likely to shed important new light into how anatomical and functional variability may contribute to neurological and psychiatric conditions and their treatment.

Glymphatic exchange contributes to the clearance of amyloid ß (Aß) and tau (Iliff et al., 2012; Iliff et al., 2014), two proteins whose mis-aggregation are believed to underlie the pathogenesis of Alzheimer’s disease (AD). Glymphatic function is impaired in rodent models of aging (Kress et al., 2014), cerebrovascular injury (Wang et al., 2017), and traumatic brain injury (Iliff et al., 2014), each of which is a risk factor for the development of Alzheimer’s-related dementia. These studies have led to the widespread supposition that impairment of glymphatic function is a key contributor to the development of age-related dementing disorders such as AD (Nedergaard and Goldman, 2020). Defining the role that glymphatic impairment may play in the development of AD and related disorders in human populations to date has been difficult to directly define. In a human post-mortem case series, participants with histopathologically-confirmed AD exhibited reduced perivascular AQP4 localization compared to cognitively-intact participants (Zeppenfeld et al., 2017). Loss of perivascular AQP4 localization was associated with Aβ and tau pathology and cognitive impairment. In a transgenic mouse model in which deletion of the alpha-1-syntrophin (*Snta1*) gene eliminates the perivascular AQP4 localization, impairment of glymphatic function and more rapid Aβ deposition was observed (Mestre et al., 2018a; Simon et al., 2022). Lastly, naturally occurring single-nucleotide polymorphisms in the human *AQP4* gene modified risk of cognitive decline in a cohort of AD patients (Burfeind et al., 2017). Although fewer studies have been conducted defining the relationship between meningeal lymphatic drainage and the development of AD-related pathology, recent studies modulating meningeal lymphatic and deep cervical lymphatic drainage demonstrate effects on both Aβ and tau pathology in rodent models of AD (Da Mesquita et al., 2018; Patel et al., 2019; Wang et al., 2019). These data from both preclinical rodent studies and human clinical studies support the role that glymphatic and meningeal lymphatic impairment plays in the development of age-related neurodegenerative disease and suggest that targeting sleep-active perivascular glymphatic exchange or wake-active meningeal lymphatic clearance may be viable approaches to the prevention and treatment of these conditions.

Skin, the largest human organ, is the setting for a peripheral lymphatic system superhighway with highly abundant lymphatic vessel network that is spatially separated into superficial and deep vascular plexuses. Much of our understanding of the anatomic and molecular details of the dermal lymphatic vasculature comes from studies of the mouse skin where the three-dimensional architecture can be visualized using fluorescence microscopy methods. These studies have revealed a hierarchy of functionally specialized vessels; the blind-ended lymphatic capillaries that take up excess interstitial fluid, cells and macromolecules, and fluid-transporting collecting vessels. The importance of functional specialization is reflected in the unique morphological features of the different lymphatic vessel types. For example, the two types of vessels display distinct organization of cell-cell junctions (Baluk et al., 2007). Other vessel type-specific features include luminal valves, lymphatic smooth muscle cells, discontinuous button junctions (Baluk et al., 2007) in lymphatic capillaries and continuous zipper junctions that are only present in the collecting lymphatic vessels (Baluk and McDonald, 2022). The use of single cell RNA sequencing can be used to define the molecular features of dermal lymphatic endothelial cells (LECs) transcriptome. Such new methodologies have allowed identification of molecularly distinct populations of lymphatic capillary, collecting vessel, and valve LECs. Molecular definition of LEC subtypes helps provide the basis for the identification of vessel-type specific markers and the creation of a vessel anatomy map. For example, lymphatic vessel endothelial hyaluronan receptor 1 (LYVE1) is a marker of lymphatic capillaries while Claudin 11 (CLND11) is expressed in lymphatic valves in the skin (Korhonen et al., 2022) but also in other organs (Takeda et al., 2019; Xiang et al., 2020; González-Loyola et al., 2021). Understanding of normal specific lymphatic vascular signatures can also help highlight the transition to lymphatic dysfunction and disease.

**FIGURE 4:**
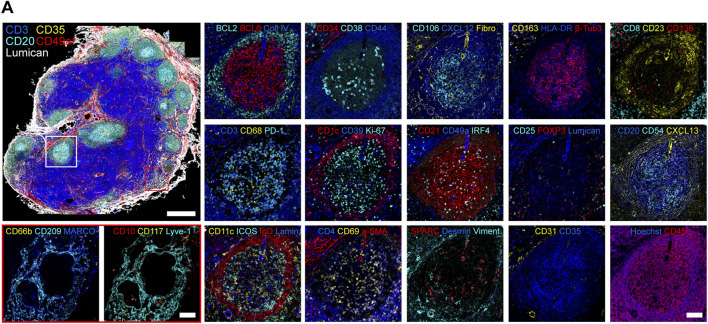
Fifty-plex confocal images of a human mesenteric lymph node obtained by the IBEX method. Two to four marker overlays for two regions (germinal center, white rectangle and medullary cords, red rectangle) are shown in higher zoom. Scale bars, 500 and 100 μm, for the overview and zoom-in images, respectively. β-Tubulin 3 (β-Tub3), collagen IV (Coll IV), fibronectin (Fibro), laminin (Lamin), and vimentin (Viment). Figure kindly provided by *Nature Methods* (Hickey et al., 2022).

Lymph nodes play a critical role in lymphatic function by filtering lymphatic fluid, generating immune responses and containing pathogens (Kastenmüller et al., 2012; Grant et al., 2020). Notably, humans have 500 to 600 lymph nodes (LNs) (Moore and Bertram, 2018), each possessing unique functional properties and cellular compositions depending on their location in the body and disease status of the patient (Carter and Collins, 1974; Mowat and Agace, 2014; Esterházy et al., 2019). To achieve these diverse functions, each individual LN acts as a functional unit subdivided into the outer cortex, paracortex, and medulla (Takeda et al., 2019). Moreover, new technologies revealed six transcriptionally distinct LEC subtypes located in specific anatomical sites in human LNs supporting the local specialization of their function. For example, LECs lining the floor of subcapsular and medullary sinuses constitutively expressed neutrophil chemoattractants, while LECs lining medullary sinuses supported the adhesion of neutrophils into the human LN medulla (Takeda et al., 2019; Xiang et al., 2020). Beyond these major tissue compartments, LNs possess several microanatomical niches that allow for the generation of robust and efficient immune responses (Grant et al., 2020). Multiplexed antibody-based imaging (Hickey et al., 2022) allows for the interrogation of tissues at single cell resolution with dozens of antibodies directed against protein biomarkers expressed by the diverse cell types and anatomical structures present in normal and diseased tissues. One such technique (Figure 4), Iterative Bleaching Extends multi-pleXity (IBEX) is a cyclic immunolabeling and chemical bleaching method that enables more than 50 parameters to be visualized *in situ* (Radtke et al., 2020; Radtke et al., 2022a)*.* Importantly, advanced sequencing and imaging technologies can be integrated to provide a molecular and spatial map of human tissues as recently shown for normal and follicular lymphoma LNs (Radtke et al., 2022c).

### Research gaps and opportunities

Emerging evidence shows that heterogeneity in the lymphatic vascular system impacts multiple physiological and pathological processes. This heterogeneity defines functional specialization of lymphatic vessels within different organs such as immune modulation, uptake of dietary fat, and clearance of cholesterol. The contributions of inflammation, fibrosis, adipose tissue deposition, and infection in the regulation of lymphatic dysfunction after an initial lymphatic insult, as in secondary lymphedema, remain to be elucidated. Potential approaches include the development of improved experimental models, such as inducible lymphatic endothelial cell ablation, to better recapitulate the human disease. Moreover, new treatments and clinical trials investigating the effect of weight-loss (Schmitz et al., 2019), anti-T helper two immunotherapy (Mehrara et al., 2021), or adenoviral delivery of VEGF-C together with lymph node transfer (NCT02994771) may also provide more critical insight. Ultimately, the identification of easily accessible, reliable biomarkers of lymphatic dysfunction would provide a valuable resource to assist not only in the conclusive diagnosis of lymphedema and other lymphatic pathologies, but also help to identify and diagnose subtle asymptomatic lymphatic alterations that might contribute to the vast array of lymphatic disease states and asymptomatic lymphatic dysfunction. With lipedema, further investigations are warranted to identify how different cells in the adipose tissue take part in the development of the disease.

Recent studies suggested a beneficial role for lymphatics in restoring heart function after cardiac injury, as it has been shown that abnormal cardiac lymph flow promotes cardiac edema, and that cardiac lymphatic vessels could be therapeutic targets to restore cardiac function after heart injury. It was reported that specific stimulation of lymphangiogenesis (growth of lymphatic vessel) by ectopic VEGF-C delivery to the infarcted heart, improves cardiac function and prevents adverse cardiac remodeling in rodent acute MI models (Cui, 2010; Henri et al., 2016; Vuorio et al., 2017; Vieira et al., 2018). However, the mechanisms by which lymphangiogenesis improves cardiac function remain to be fully elucidated, and whether lymphatics could play additional functions during heart regeneration is not yet known. Results about lymphoangiocrine factors/Reelin in cardiac development and repair (Liu et al., 2023) could become a valuable resource to identify unique novel therapeutic approaches for the treatment and prevention of cardiac disorders. In particular, determining how cardiac lymphatic vessels in general, and Reelin in particular contribute to enhanced cardio-protection after an infarct could lead to the development of novel therapies for the alleviation of several cardiac pathological conditions. This knowledge could also lead to the use of novel diagnostic tools to help identify lymphatic associated cardiac anomalies.

Understanding the role of lymphatics in the central nervous system is a burgeoning field with endless opportunities. Certain areas for future research would include understanding the anatomical and function linkage between distributed perivascular pathways and meningeal routes of lymphatic drainage from the cranium particularly in the human brain. Moreover, further delineation of the common or distinct signaling and physiological processes regulating sleep-active glymphatic function and meningeal lymphatic drainage is needed. The relative contribution of glymphatic-lymphatic function to the clearance of different pathogenic proteins in humans is unknown, as well as how these processes change in the setting of neurodegenerative diseases like AD. Finally, the contribution of lymphatic biology and its dysfunction to neurological and psychiatric conditions beyond neurovascular disorders and neurodegenerative diseases remains almost entirely unexplored.

Notably, genes identified through single cell level analyses are likely not only markers of cell subtype identity, but functionally important and thus pointing to potential biomarkers and targets for future studies and interventions. For instance, changes in the LEC transcriptome during pathological changes, or differences between the normal LEC transcriptome from various tissues are likely to reveal state and organ-specific molecular features that could be used for targeting various parts of the lymphatic system.

Many single cell resolution studies provide detailed assessments of murine lymphoid organs, however, the discrete tissue structures of human LNs remain an open area of exploration. LNs serve as the staging ground of innate and adaptive immune responses (Kastenmüller et al., 2012) and host a variety of cells that require a multitude of markers for comprehensive profiling. To this end, a recent report estimated a representative human LN to contain 34 anatomical structures, 45 cell types, and 223 protein biomarkers (Börner et al., 2021). Although ambitious in scope, the current Anatomical Structure and Cell Types plus Biomarkers table of the Human Reference Atlas, which is a collaborative project to map all the cells of the human body to advance biomedical research, aims to connect the three-dimensional representations of anatomy (Börner et al., 2021). The table for the LN is very much a living publicly available document to be expanded upon by using emerging technologies and data from new studies^3^. Remaining challenges for the field include accurate segmentation of irregularly shaped cells and deep knowledge of ill-defined cell types such as fibroblastic reticular cells. Fortunately, recent progress has been made on quantifying stromal cell types in human LNs by single cell RNA-sequencing and multiplexed imaging (Jalkanen and Salmi, 2020; Abe et al., 2022; Radtke et al., 2022a; Radtke et al., 2022c).

Gene expression differences in closely related cell subtypes are often quantitative rather that qualitative, showing gradual changes along an anatomical axis, or even heterogeneity among similar cells within the same anatomical structure, such as a specific blood or lymph vessel subtype. Thus, an open question in the field of cell marker genes is distinguishing been the detection of same cell subtypes vs. identifying different cell types. In addition, the transcriptome may not always reflect protein expression, thus validation of marker gene expression in the tissue is critical for establishing the identified gene expression profiles. A detailed visualization of the three-dimensional blood and lymphatic vasculature within human tissues, including skin and LNs, remains challenging (Currlin et al., 2022). Construction of a human body biomolecular atlas across the body scales requires extensive knowledge of its cellular composition and tissue architecture (Regev et al., 2017; Snyder et al., 2019).

The National Institutes of Health (NIH) is seeking to turn discovery into health. Many open resources are available to help advance the knowledge of normal human biology and function through the application of new technologies and to identify the underpinnings of various diseases, including lymphatic disease (Radtke et al., 2022b).^3^ As one of these efforts, the NIH Common Fund’s Human Biomolecular Atlas Program (HuBMAP)^4^ has supported single cell resolution mapping of various normal organs, including lymphatic organs such as the spleen, thymus, and lymph node (Snyder et al., 2019), as well as organizing and harmonizing the large amount of information arising from this and other similar efforts, by supporting and leading the development of an open Human Reference Atlas that captures the three-dimensional spatial properties of 26 human organs and the names of their anatomical structures and cell types, and the biomarkers (e.g., genes, proteins, lipids) (Börner et al., 2021).

A draft table of anatomical structures, cell types, and biomarkers of several lymphatic organs, but not including all of the components of the lymphatic system, has been published (Börner et al., 2021) and is updated regularly^5^. Notably, still missing from the efforts to map the human lymphatic system is a map of its lymphatic vasculature. The efforts to create a body-wide map of the human lymphatic vasculature from the anatomical down to single cell level are critical to organizing what we already know and identifying what is still unknown, so that critical knowledge gaps can be filled. Further, making this information available will reduce barriers for researchers and clinicians to study, question, and innovate. Ultimately, these efforts will provide solutions for patients suffering from lymphatic and lymphatic-related diseases.

## Summary

Advancement of lymphatic research requires refinement of existing knowledge and application of advanced spatial mapping approaches for a greater understanding of the normal lymphatic system and connections with development of lymphatic dysfunction and disease.

Working together across specialty domains and across the human body scales we hope to further expand this knowledge base for the betterment of the human condition.

## Footnotes

1
https://miro.com/app/board/o9J_l_LnfKg=/

2 Griffin Weber; Sujin Lee; Rajeev Malhotra. HuBMAP ASCT+B Tables. Blood Vasculature v1.2, https://doi.org/10.48539/HBM954.HNGT.899.

3
https://zenodo.org/record/6629388#.YzMhI3bMIuV

4
https://commonfund.nih.gov/hubmap

5
https://hubmapconsortium.github.io/ccf/pages/ccf-anatomical-structures.html
